# An effective processing pipeline for harmonizing DNA methylation data from Illumina’s 450K and EPIC platforms for epidemiological studies

**DOI:** 10.1186/s13104-021-05741-2

**Published:** 2021-09-08

**Authors:** Lauren A. Vanderlinden, Randi K. Johnson, Patrick M. Carry, Fran Dong, Dawn L. DeMeo, Ivana V. Yang, Jill M. Norris, Katerina Kechris

**Affiliations:** 1 Department of Biostatistics and Informatics, Colorado School of Public Health, University of Colorado Anschutz Medical Campus, Aurora, CO USA; 2 Department of Epidemiology, Colorado School of Public Health, University of Colorado Anschutz Medical Campus, Aurora, CO USA; 3 Channing Division of Network Medicine, Department of Medicine, Brigham and Women’s Hospital and Harvard Medical School, Boston, MA USA; 4 School of Medicine, University of Colorado, Anschutz Medical Campus, Aurora, CO USA

**Keywords:** DNA methylation, Illumina 450K, Illumina EPIC, Platform harmonization

## Abstract

**Objective:**

Illumina BeadChip arrays are commonly used to generate DNA methylation data for large epidemiological studies. Updates in technology over time create challenges for data harmonization within and between studies, many of which obtained data from the older 450K and newer EPIC platforms. The pre-processing pipeline for DNA methylation is not trivial, and influences the downstream analyses. Incorporating different platforms adds a new level of technical variability that has not yet been taken into account by recommended pipelines. Our study evaluated the performance of various tools on different versions of platform data harmonization at each step of pre-processing pipeline, including quality control (QC), normalization, batch effect adjustment, and genomic inflation. We illustrate our novel approach using 450K and EPIC data from the Diabetes Autoimmunity Study in the Young (DAISY) prospective cohort.

**Results:**

We found normalization and probe filtering had the biggest effect on data harmonization. Employing a meta-analysis was an effective and easily executable method for accounting for platform variability. Correcting for genomic inflation also helped with harmonization. We present guidelines for studies seeking to harmonize data from the 450K and EPIC platforms, which includes the use of technical replicates for evaluating numerous pre-processing steps, and employing a meta-analysis.

**Supplementary Information:**

The online version contains supplementary material available at 10.1186/s13104-021-05741-2.

## Introduction

Numerous epidemiological studies have examined DNA methylation due to its important role in physiological processes, and the development and progression of human diseases [1]. Microarrays are widely used for DNA methylation profiling, and are affordable for studies with large sample sizes. Illumina’s methylation array is a common choice in many data repositories such as TCGA with ~ 12,000 samples, ENCODE with ~ 250 datasets and GEO with ~ 7000 datasets (April 2020).

DNA methylation array technologies have evolved so more individual methylation CpG sites can be evaluated on a single array. Illumina’s BeadChip methylation microarrays are extremely popular, the most recent being the HumanMethylationEPIC BeadChip (“EPIC”) released in 2016 that measures ~ 8,50,000 CpG sites (probes), which is an increase from the previous array (HumanMethylation450K BeadChip, “450K”). In many studies, both platforms have been used, due to technology updates in the middle of large projects, or multiple batches for studies over time [2–6]. Some investigators are interested in maximizing sample size for research questions by analyzing data from the current EPIC and older 450K arrays. Numerous publications focus on certain aspects of pre-processing such as normalization [7] or probe filtering [8] but there are currently no papers that consider the entire pipeline. Establishing best practices is relevant for other epidemiological studies that needed to change platforms mid-study, in addition to the re-analyses of public data.

We evaluate the performance of common harmonization tools of 450K and EPIC data at various pre-processing and analytical steps using the Diabetes Autoimmunity Study in the Young (DAISY), which prospectively follows genetically high-risk children for the development of type 1 diabetes (T1D) [4]. We explored normalization, probe-level QC and filtering, batch effect adjustment, and genomic inflation by testing methods that were easy to implement from well-established and documented R packages. Finally, we provide evaluation guidelines for studies facing similar harmonization challenges.

## Main text

### Methods

**Fig. 1:**
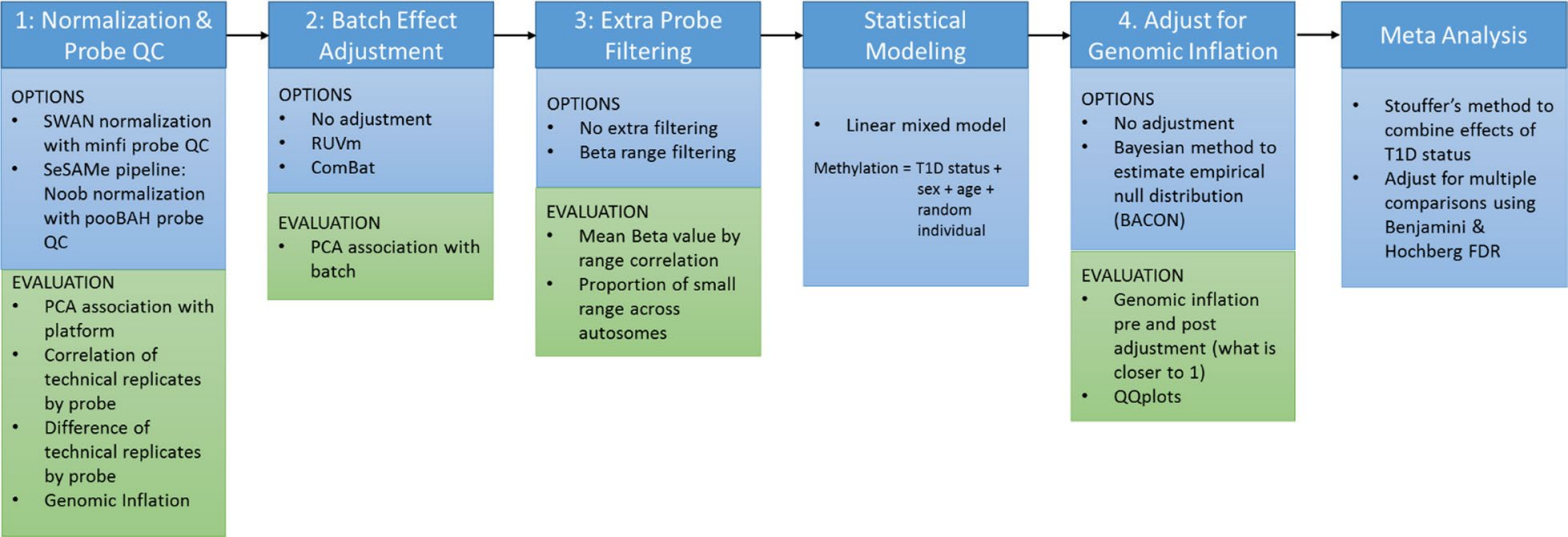
Pipeline Methods Considered. The four main pre-processesing steps are: 1. Normalization and probe QC, 2. Batch effect adjustment, 3. Extra probe filtering and 4. Genomic inflation adjustment. The various methods considered for each step is listed along with the evaluation(s) used to assess these methods

Figure 1 shows a summary of the pre-processing pipeline and the data harmonization evaluations.

#### Data

Peripheral whole blood was collected prospectively from individuals enrolled in DAISY. Cases of T1D were frequency matched to controls, and DNA methylation generated on up to five prospective samples per subject [4]. The EPIC platform replaced the 450 K during data acquisition. There were 42/42 and 45/45 matched T1D cases/controls (corresponding to 184 and 211 unique arrays) for the 450 K and EPIC platforms, respectively.

#### Normalization and probe-level QC

First, three established normalization and probe-level QC methods were evaluated (Fig. 1): subset-quantile within array normalization (SWAN, [9]), normal-exponential using out-of-band probes (Noob, [10]) and single-sample Noob (ssNoob,[11]). SWAN and ssNoob normalizations were performed within the minfi package [12], while Noob normalization was performed in the SeSAMe package [7]. We examined two detection above background methods: minfi’s default [12] and SeSAMe’s pooBAH [7] and coupled it to the normalization in the same R package. Filtering on probe-level QC was performed after each normalization prior to evaluation of platform effects and included removing probes with known SNPs in the probe sequence [13] as well as cross-reactive probes [14]. See Jonhson et al. for full detail [4].

For evaluating normalization and probe-level QC procedures, we looked at the first 10 principal components (PCs) to determine if there was a large platform effect across components, as well as the three technical replicate metrics mentioned below. For the PCA, all probes that passed QC were included, regardless if present or not on the other platform, excluding probes on chromosome X.

Twelve technical replicates were selected to be balanced with respect to sex, age, and islet autoimmunity (IA) status. To examine this data we used three metrics: (1) a difference in methylation Beta value at the individual probe (Additional file 1: Eqn S1), (2) correlation of all probes across a single technical replicate pair (Additional file 1: Eqn S2) and (3) correlation of the technical replicate pairs across a single probe (Eqn S3, see Additional file 1: Methods).

#### Batch effect adjustment

Second, we applied two different within platform batch effect methods: ComBat [15] and RUVm [16] (Fig. 1) in the sva (v3.30.0) and missMethyl (v1.16.0) packages respectively.

#### Additional probe filtering

Third, we explored supplemental probe filtering by removing low range probes (< 5% Beta) as suggested [8] and compared how well technical replicates correlated.

#### Statistical analysis

We performed a linear mixed model using T1D status to predict methylation (M-values) while adjusting for age and sex and using subject as a random effect using the R/nlme package (v3.1–137) [17].

#### Genomic inflation

We corrected for genomic inflation using the R/BACON package (v1.10.1) [18]. In brief, BACON estimates an empirical null distribution using a Bayesian method to account for the bias and inflation of test-statistics specific to EWAS datasets.

#### Meta-analysis

Stouffer’s meta-analysis method [19] combined the statistical results from the two different platforms. This method generates a single meta-analysis p-value for each probe, and accounts for consistent effect direction.

### Results

For each of the processing Steps 1–4, we compared different options with a variety of data harmonization evaluation diagnostics (Fig. 1).

#### Normalization evaluation

**Fig. 2:**
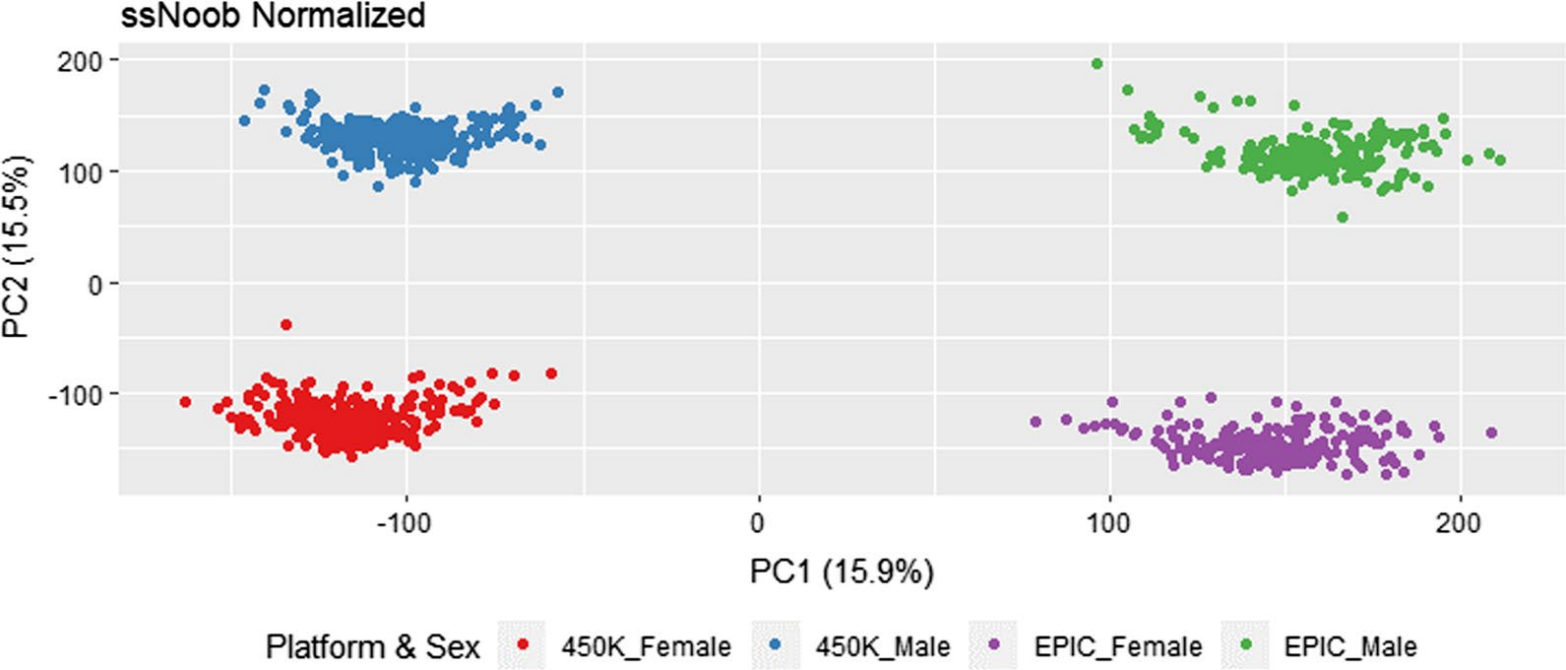
Platform Effect. The 1st and 2nd principal components (PCs) from the ssNoob normalization are plotted with colors symbolizing both platform and sex. Red and blue dots signify the 450K platform while purple and green dots signify the EPIC data. Red and purple dots signifiy females and blue and green dots signify males. Percent variance explained by each PC is noted in parentheses

First, we explored normalization of both datasets together using ssNoob (coupled with minfi probe QC), as recommended by Fortin [11]. After associating the first 10 PCs with platform, we found the first and second PC had extremely high associations with platform and sex respectively (Fig. 2). Sex differences are expected to be a large contributor to methylation profiles as methylation is known to have a large role in female X-chromosome inactivation. Applying subsequent batch adjustment did not reduce the strong platform effect (Additional file 1: Table S1), regardless of method applied. Therefore, we applied normalization procedures by each platform separately.

**Fig. 3:**
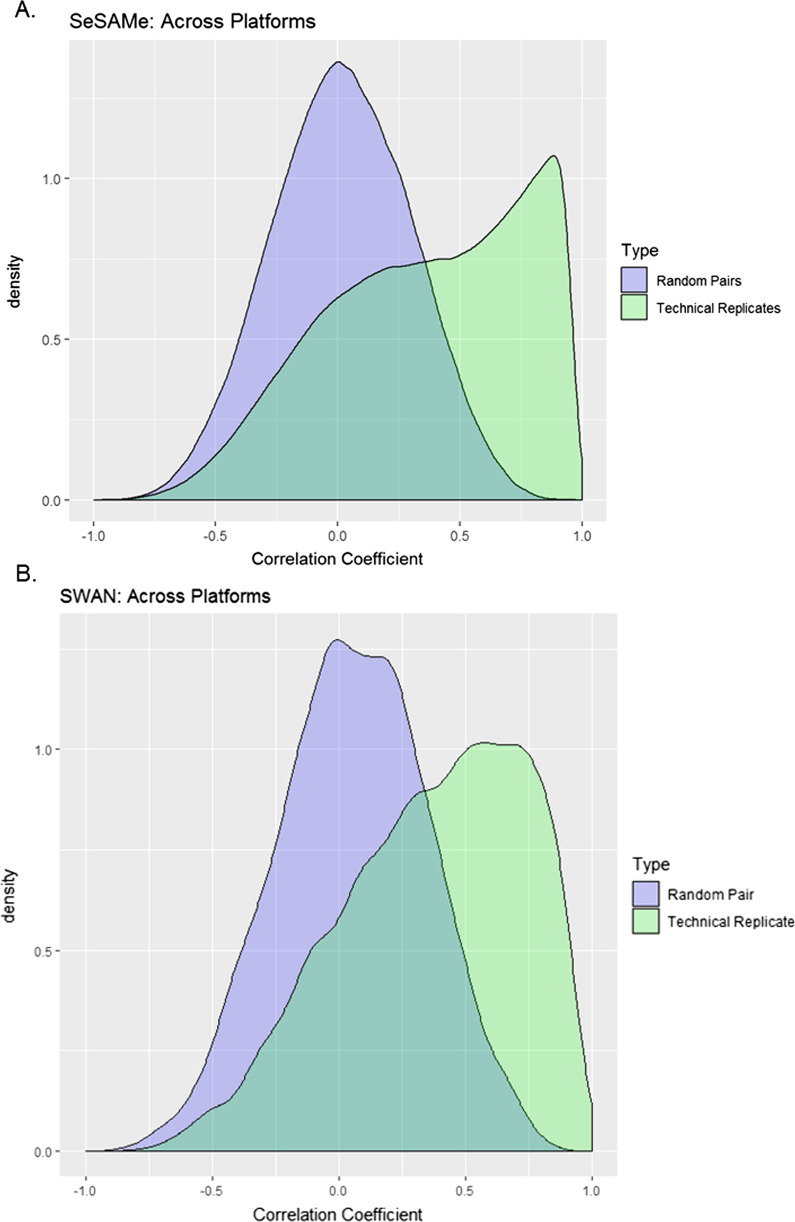
Correlation of Technical Replicates. Density plots of correlations across the platform technical replicates for each probe (n = 12, green) as well as a random subset of pairs for comparison (n = 12, purple) for the data normalized using **A** SeSAMe and **B** SWAN. The median correlation coefficient among technical replicates is both 0.41 in the SeSAMe and SWAN methods. The 1st and 3rd quartiles for technical replicates for SeSAMe and SWAN were (0.06, 0.72) and (0.11, 0.67) respectively

To explore the effect of SWAN or SeSAMe on harmonization of platforms, we examined technical replicates across platforms. Correlation across probes for each pair of technical replicates (Additional file 1: Eqn S2) was extremely high (> 0.98) for both methods. This is not surprising given the large amount of probes used to calculate each correlation, and similar to high correlation between random pairs of samples (> 0.97). Individual probe correlations deemed much more informative (Eqn S3). We generated densities of probe-level correlations across the technical replicates as well as across random samples (Fig. 3). The distribution of the random sample correlations for both the SeSAMe and SWAN are centered around 0 and look more Gaussian compared to the distributions for the technical replicate correlations, which look like a mixture of two or more distributions in addition to being centered around a higher correlation coefficient.

We examined the absolute differences in methylation on the probe-level (Additional file 1: Eqn S1) of the Beta value (% methylation) for each technical replicate (Additional file 1: Figure S1). In all technical replicate pairs, SeSAMe has a tighter distribution closer to 0 and shown to harmonize better, while SWAN has higher absolute differences. We report the mean bias (and 95% CI) for the differences between platforms by normalization type (Additional file 1: Table S2). Given these results, we moved forward with the SeSAMe normalization (see Additional file 1: for probe QC filtering numbers).

#### Batch effect adjustment

Even normalizing within each platform type, we still have technical batch effects to consider, since a variety of factors can add unwanted technical variation [20]. Therefore, we examined within platform batch effect being defined as plate and row location combination. We performed ComBat and RUVm to adjust for within platform batch effects on the SeSAMe dataset. Based on the PC analysis, ComBat performed slightly better, as the top PCs were less associated with batches defined as plate by rows (Additional file 1: Figure S2, [20]). Our results of ComBat outperforming other methods is consistent with Jiao and colleagues [21].

#### Probe filtering

Applying the Logue beta range filteer [8], removing probes with < 5% Beta methylation range, resulted in removing 15.8% (59,397) and 33.9% (225,342) of probes in the 450 K and EPIC platforms respectively. The mean beta values for the probes which were removed fell only on the extremes for both platforms (Additional file 1: Figure S3), while the probes which passed this criteria had mean beta values throughout the potential 0–100% methylation values. Additional considerations are summarized in the Supplement.

#### Genomic inflation in statistical analysis

After statistical analysis, it’s important to consider the genomic inflation factor lambda (i.e., general inflation of test statistics due to population structure), which is the ratio of the median of the observed distribution of the test statistics to the expected median, and should be close to 1. In the 450 K platform, the SWAN normalized dataset resulted in an extreme genomic inflation whereas the EPIC was deflated (Additional file 1: Figure S6). However, genomic inflation was comparable between the platforms for the SeSAMe normalized datasets. To account for any additional genomic inflation we applied BACON [18], which was developed to control for genomic inflation specifically for EWAS. After this adjustment, the genomic inflation factor for the SeSAMe 450 K and EPIC datasets were 1.03 and 1.08 respectively.

To perform the meta-analysis, we only kept probes present in both the SeSAMe datasets (1,99,243 probes). Final results are reported by R. K. Johnson and colleagues [4]. This final pipeline as it gives comparable candidate probes to other DNA methylation papers in T1D [22–25].

### Discussion

Pre-processing any ‘omics dataset including Illumina’s BeadChip array can have substantial effects on downstream analyses. The introduction of an updated array adds the additional hurdle of harmonizing more than one platform to leverage all available data. If possible, we recommend including technical replicates in the study design to aid in assessing the quality of pre-processing steps as it was key for our harmonization evaluation process of the various methods. Additional file 1: Figure S7 summarizes our recommended best practices based on the tested approaches. We realize new methods are constantly evolving in this field, and this flow chart aims to help guide analysts in what decisions need to be made throughout this process.

There are special considerations regarding probe filtering, which is performed at two stages. The first stage is based on low quality probes identified after normalization, and the second stage is before statistical modeling based on removing non-varying probes. Other filtering criteria such as probes with both high variance and high differences in beta values between technical replicates should be considered. In the first probe filtering, the pooBAH method (part of SeSAMe pipeline) removed a high number of probes compared to SWAN, specifically those on sex chromosomes. Other normalization procedures, such as functional normalization [26], which utilize control probes were not reported, but may work well for some datasets. However, the resulting genomic inflation values were more consistent among the platforms and closer to one, which is desired. This suggests pooBAH correctly identifies germline and somatic deletions that would be causing this inflated signal. However, the use of pooBAH filtering on sex chromosomes should be considered with caution.

Another consideration is adjusting for cell type proportion and for this specific analysis it is discussed in depth in Johnson et al. 2020 [4]. There are conflicting viewpoints for whether to include cell type adjustment [27, 28], and should depend on the specific study design.

In addition to a meta-analysis, we explored an alternative approach, where data were pooled into one statistical model and included a fixed covariate of platform and subsequently adjusted for genomic inflation afterward. In this study, results of the single model were qualitatively similar to the meta-analysis and provided little evidence to support one method over the other. We recommend that both approaches be considered, in addition to random effect methods [29].

In summary, our evaluation methods relied on technical replicates, which we highly recommend. The harmonization evaluation metrics on the technical replaces were used to compare methods at different steps, and can be used to evaluate other options as new methods are developed. We hope our guidelines aid others in their endeavors for performing analyses consisting of both 450 K and EPIC platforms.

## Limitations

Others have explored individual steps in this pipeline [8, 11], therefore we did not examine individual steps in depth using multiple datasets or simulations. The goal of this work was to evaluate the entirety of steps involved in a methylation processing pipeline based on data from both Illumina’s 450 K and EPIC platforms and how it affects harmonization. We do not claim that our recommended pipeline will be best in all scenarios, but illustrate what factors need to be considered for selecting a pipeline with other datasets, and new methods as they are published.

## Supplementary Information

Additional file 1: **Table S1.** PC Association with Platform. Principal component analysis was performed across the ssNoob normalized dataset (e.g. both the 450K and EPIC platforms normalized together) with and without batch effect adjustment using Combat. For the first 10 PCs, the percent variance explained and the p-value for representing the association between the PC and platform is reported. Associations with a p-value < 0.05 are highlighted in yellow. **Figure S1.** Technical Replicate Differences. Density plots of the difference in methylation (Beta value) between pairs of technical replicates for each platform (EPIC or 450K) for the two methods (SeSAMe in blue and SWAN in red). Each plot displays one of the twelve pairs of technical replicates. **Figure S2.** Batch Effect Adjustment. Heatmaps of the association between principal components and batch for both the unadjusted raw normalized data, the RUVm adjusted data and ComBat adjusted data in both the A. 450K and B. EPIC platforms. **Figure S3.** Mean Beta value for probes with no variability. Histograms showing the mean Beta value for those probes which failed the range filter (Beta range < 0.05) are shown for the A. 450K and B. EPIC platforms. Histograms for the mean Beta value for those probes which passed the range filter (Beta range > 0.05) are shown for the C. 450 and D. EPIC platforms. **Figure S4.** Effect of extra filtering by probe variability on genomic distribution of probes. The proportion of probes on each chromosome is shown in the pre- and post- probe range filtering datasets in red and blue respectively (filtered probes with a Beta range < 0.05) for both the A. 450 K and B. EPIC platforms. **Figure S5.** Technical Replicate Correlation and Beta Range. Probe correlation coefficients from the technical replicates within the 450 K (Eq-2) is plotted against the methylation Beta range. **Figure S6.** Genomic inflation factor across different datasets. The qq-plots for the different datasets are shown for A. SWAN normalized 450K (lambda = 3.02), B. SWAN normalized EPIC (lambda = 0.83)., C. SeSAMe 450K (lambda = 0.93) and D. SeSAMe EPIC (lambda = 0.98). The blue dots are the observed p-values, while the black line shows the expected distribution these p-values should follow. **Figure S7.** Final meta-analysis pipeline. The final recommendations for a meta-analysis using the two Illumina methylation platforms. The blue box represents the raw data, the orange boxes represent each processing step, the gray boxes report how many probes are filtered out in each step and the green boxes are the final methylation candidates.

## Data Availability

The datasets generated during and/or analysed during the current study are accessible through GEO Series accession number GSE142512 (https://www.ncbi.nlm.nih.gov/geo/query/acc.cgi?acc=GSE142512). All data generated for this study are included in Johnson R.K. and colleagues [4]. This manuscript was based on the preprocessing for the analyses used in the Johnson R.K. publication [4].

## Abbreviations

QC: Quality control
DAISY: Diabetes Autoimmunity Study in the Young
T1D: Type 1 diabetes
SWAN: Subset-quantile within array normalization
Noob: Normal-exponential using out-of-band probes
ssNoob: Single-sample Noob
IA: Islet autoimmunity
PC: Principal component

## References

[1] Jin Z, Liu Y (2018). DNA methylation in human diseases. Genes Dis.

[2] Abdulrahim JW, Coulter KL, Elizabeth G, Siegler IC, Redford W, Ravi K (2019). Epigenome-Wide Association Study for All-Cause Mortality in a Cardiovascular Cohort Identifies Differential Methylation in Castor Zinc Finger 1 (CASZ1). J Am Heart Assoc.

[3] Fernandez-Jimenez N, Allard C, Bouchard L, Perron P, Bustamante M, Bilbao JR (2019). Comparison of Illumina 450K and EPIC arrays in placental DNA methylation. Epigenetics.

[4] Johnson RK, Vanderlinden LA, Dong F, Carry PM, Seifert J, Waugh K (2020). Longitudinal DNA methylation differences precede type 1 diabetes. Sci Rep.

[5] McEwen LM, Jones MJ, Lin DTS, Edgar RD, Husquin LT, MacIsaac JL (2018). Systematic evaluation of DNA methylation age estimation with common preprocessing methods and the Infinium MethylationEPIC BeadChip array. Clin Epigenetics.

[6] Solomon O, MacIsaac J, Quach H, Tindula G, Kobor MS, Huen K (2018). Comparison of DNA methylation measured by Illumina 450K and EPIC BeadChips in blood of newborns and 14-year-old children. Epigenetics.

[7] Zhou W, Triche TJ, Laird PW, Shen H (2018). SeSAMe reducing artifactual detection of DNA methylation by Infinium BeadChips in genomic deletions. Nucleic Acids Res.

[8] Logue MW, Smith AK, Wolf EJ, Maniates H, Stone A, Schichman SA (2017). The correlation of methylation levels measured using Illumina 450K and EPIC BeadChips in blood samples. Epigenomics.

[9] Maksimovic J, Gordon L, Oshlack A (2012). SWAN: Subset-quantile Within Array Normalization for Illumina Infinium HumanMethylation450 BeadChips. Genome Biol.

[10] Triche TJ, Weisenberger DJ, Van Den Berg D, Laird PW, Siegmund KD (2013). Low-level processing of Illumina Infinium DNA Methylation BeadArrays. Nucleic Acids Res.

[11] Fortin J-P, Triche TJ, Hansen KD (2017). Preprocessing, normalization and integration of the Illumina HumanMethylationEPIC array with minfi. Bioinforma Oxf Engl.

[12] Aryee MJ, Jaffe AE, Corrada-Bravo H, Ladd-Acosta C, Feinberg AP, Hansen KD (2014). Minfi: a flexible and comprehensive Bioconductor package for the analysis of Infinium DNA methylation microarrays. Bioinforma Oxf Engl.

[13] Maksimovic J, Gagnon-Bartsch JA, Speed TP, Oshlack A (2015). Removing unwanted variation in a differential methylation analysis of Illumina HumanMethylation450 array data. Nucleic Acids Res.

[14] Chen Y, Lemire M, Choufani S, Butcher DT, Grafodatskaya D, Zanke BW (2013). Discovery of cross-reactive probes and polymorphic CpGs in the Illumina Infinium HumanMethylation450 microarray. Epigenetics.

[15] Maksimovic J, Gagnon-Bartsch JA, Speed TP, Oshlack A (2015). Removing unwanted variation in a differential methylation analysis of Illumina HumanMethylation450 array data. Nucleic Acids Res.

[16] Davidian M, Giltinan DM (2003). Nonlinear models for repeated measurement data: an overview and update. J Agric Biol Environ Stat.

[17] van Iterson M, van Zwet EW, Heijmans BT, BIOS Consortium (2017). Controlling bias and inflation in epigenome- and transcriptome-wide association studies using the empirical null distribution. Genome Biol.

[18] Stouffer SA, Suchman EA, Devinney LC, Star SA, Williams Jr. RM. The American soldier: Adjustment during army life. (Studies in social psychology in World War II), Vol. 1. Oxford, England: Princeton Univ. Press; 1949. xii, 599 p. (The American soldier: Adjustment during army life. (Studies in social psychology in World War II), Vol. 1).

[19] Price EM, Robinson WP (2018). Adjusting for batch effects in DNA methylation microarray data, a lesson learned. Front Genet.

[20] Jiao C, Zhang C, Dai R, Xia Y, Wang K, Giase G (2018). Positional effects revealed in Illumina methylation array and the impact on analysis. Epigenomics.

[21] Belot M-P, Nadéri K, Mille C, Boëlle P-Y, Benachi A, Bougnères P (2017). Role of DNA methylation at the placental RTL1 gene locus in type 1 diabetes. Pediatr Diabetes.

[22] Disanto G, Vcelakova J, Pakpoor J, Elangovan RI, Sumnik Z, Ulmannova T (2013). DNA methylation in monozygotic quadruplets affected by type 1 diabetes. Diabetologia.

[23] Rakyan VK, Beyan H, Down TA, Hawa MI, Maslau S, Aden D (2011). Identification of type 1 diabetes-associated DNA methylation variable positions that precede disease diagnosis. PLoS Genet.

[24] Stefan M, Zhang W, Concepcion E, Yi Z, Tomer Y (2014). DNA methylation profiles in type 1 diabetes twins point to strong epigenetic effects on etiology. J Autoimmun.

[25] Fortin J-P, Labbe A, Lemire M, Zanke BW, Hudson TJ, Fertig EJ (2014). Functional normalization of 450k methylation array data improves replication in large cancer studies. Genome Biol.

[26] Barton SJ, Melton PE, Titcombe P, Murray R, Rauschert S, Lillycrop KA (2019). In Epigenomic studies, including cell-type adjustments in regression models can introduce multicollinearity, resulting in apparent reversal of direction of association. Front Genet.

[27] Titus AJ, Gallimore RM, Salas LA, Christensen BC (2017). Cell-type deconvolution from DNA methylation: a review of recent applications. Hum Mol Genet.

[28] Willer CJ, Li Y, Abecasis GR (2010). METAL: fast and efficient meta-analysis of genomewide association scans. Bioinformatics.

[29] Viechtbauer W (2010). Conducting meta-analyses in R with the metafor Package. J Stat Softw.

