# Supplementary material for: An effective processing pipeline for harmonizing DNA methylation data from Illumina’s 450K and EPIC platforms for epidemiological studies
Source: BMC Res Notes. 2021 Sep 8;14:352. doi: 10.1186/s13104-021-05741-2 (PMC8424820; doi:10.1186/s13104-021-05741-2)

**Supplementary Methods**

*Data*

The DAISY dataset includes 687 total arrays for 256 unique subjects on the 450K, and 475 total arrays for 178 unique subjects on the EPIC. For quality control, these included replicates of 12 samples across platforms and replicates of 32 samples within the 450K platform (Johnson et al., 2020). These data were generated to answer numerous scientific hypotheses using a combination of outcomes and time points. Accordingly, the DNA methylation pre-processing and QC steps were conducted on the full set of samples to produce a final dataset for use in all subsequent analyses.

*Batch Effects*

We considered batch effect as the plate and row position combination. In total there were 15 plates (nine on the 450K and six on the EPIC). By Illumina’s design, 450K has six different row position possibilities, while the EPIC has eight. We included row position as part of the batch effect due to previous studies finding positional effects (Jiao et al., 2018).

*Normalization and Probe-level QC*

The specific combination of Noob normalization and pooBAH probe QC filtering will be referred to as SeSAMe (Zhou et al., 2018) as this is the recommended normalization and probe QC combination in the SeSAMe pipeline. SWAN and SeSAMe were performed on each platform individually, while ssNoob was performed on both platforms together (with only probes on the EPIC that were also present on the 450K) as suggested by Fortin (Fortin et al., 2017).

When examining the two different detection above background methods: minfi’s default (Aryee et al., 2014) and SeSAMe’s pooBAH (Zhou et al., 2018), we coupled it to the normalization which was in the same R package for ease of coding execution. In regards to the different detection above background methods, the probe QC in minfi (utilized in the SWAN method) assumes the fluorescence intensity is normally distributed and a parametric method is used to determine if the fluorescence intensity detected from the probe expressed significantly higher than the negative control probes. In contrast, the pooBAH method uses an empirical cumulative distribution function and does not assume the fluorescence intensity is distributed normally.

Technical replicate metrics used for evaluation:

For looking at differences between technical replicates at the probe level we used:

$\Delta_{r,i}= X_{r, i}^{450K}- X_{r, i}^{EPIC}$ (S1)

Where: $X$ is the methylation Beta value (methylation Beta value is the ratio of the methylated signal intensity to the total methylated and unmethylated signal intensity).

$r$ = 1…R are the technical replicates

$i$ = 1… P are the number of CpG sites

450K and EPIC represent the 2 platforms and R = 12 in our example.

We also looked at the correlations between the technical replicates:

$\rho_{r}=corr(\vec{X}_{r}^{450K}, \vec{X}_{r}^{EPIC})$ (S2a)

$\rho_{r}=\frac{\sum_{i=1}^{p} ( X_{r, i}^{450K}- \bar{X}_{r}^{450K})(X_{r, i}^{EPIC}- \bar{X}_{r}^{EPIC})}{\sqrt{\sum_{i=1}^{p} {( X_{r, i}^{450K}- \bar{X}_{r}^{450K})}^{2}}\sqrt{\sum_{i=1}^{p} (X_{r, i}^{EPIC}- \bar{X}_{r}^{EPIC})^{2}}}$ (S2b)

Where $i$ and $r$ are denotes as above in equation (1). $X$ is the methylation M-value and the bar symbol denotes the mean over the $i^{th}$ CpG site.

Correlation of CpG sites across technical replicates was also examined:

$\rho_{i}=corr(\vec{X}_{i}^{450K}, \vec{X}_{i}^{EPIC})$ (S3a)

$\rho_{i}=\frac{\sum_{r=1}^{R} ( X_{r, i}^{450K}- \bar{X}_{i}^{450K})(X_{r, i}^{EPIC}- \bar{X}_{i}^{EPIC})}{\sqrt{\sum_{r=1}^{R} {( X_{r, i}^{450K}- \bar{X}_{i}^{450K})}^{2}}\sqrt{\sum_{i=1}^{R} (X_{r, i}^{EPIC}- \bar{X}_{i}^{EPIC})^{2}}}$ (S3b)

Where $X$, $i$ and $r$ are denoted as above in the previous correlation. The bar symbol denotes the mean over the $r$ technical replicates.

**Supplementary Results**

*Probe QC at the Normalization Step*

It should be noted after probe QC performed in conjunction with a normalization procedure, the number of probes which failed QC using the SeSAMe method was much higher than the SWAN method. Of the original 484,976 probes on the 450K, we were left with 375,020 and 454,078 probes for SeSAMe and SWAN methods respectively. Of the 866,836 probes on the EPIC, we were left with 664,615 and 833,798 probes for the SeSAMe and SWAN methods respectively.

*Probe Filtering*

The proportion of probes on the autosomes were very similar pre and post this range filtering in both the 450K and EPIC platforms (Supplement Figure S4) showing this filtering method does not affect one area of the genome more than others. We also show the higher probe-level technical replicate correlation within platform (Eqn – S3) is associated with those probes with higher methylation Beta range across all samples (Supplemental Figure S5).

**Supplementary Discussion**

We did remove a substantial amount of probes using the additional methylation Beta range filtering suggested by Logue et al (Logue et al., 2017). Since neither the 450K nor EPIC array is specifically designed for our population of interest, we expect many probes to be either completely methylated or unmethylated and not vary across subjects.

**Supplemental Tables & Figures**

**Supplemental Table S1: PC Association with Platform.** Principal component analysis was performed across the ssNoob normalized dataset (e.g. both the 450K and EPIC platforms normalized together) with and without batch effect adjustment using Combat. For the first 10 PCs, the percent variance explained and the p-value for representing the association between the PC and platform is reported. Associations with a p-value < 0.05 are highlighted in yellow.

**Supplemental Table S2: Platform Bias by Normalization Procedure.** The mean bias (the mean difference between 450K and EPIC array) is reported for each technical replicate pair along with the 95% CI of that bias for either the SeSAMe or SWAN normalization pipeline.

| **Technical Replicate Pair** | **SeSAMe**  **Mean Bias** | **SeSAMe**  **95% CI** | **SWAN**  **Mean Bias** | **SWAN**  **95% CI** |
| --- | --- | --- | --- | --- |
| TR1 | 0.032 | (-0.053, 0.117) | 0.048 | (-0.061, 0.157) |
| TR2 | 0.029 | (-0.050, 0.109) | 0.046 | (-0.058, 0.150) |
| TR3 | 0.034 | (-0.060, 0.128) | 0.050 | (-0.070, 0.169) |
| TR4 | 0.024 | (-0.053, 0.102) | 0.039 | (-0.063, 0.141) |
| TR5 | 0.029 | (-0.073, 0.131) | 0.044 | (-0.076, 0.164) |
| TR6 | 0.021 | (-0.061, 0.103) | 0.037 | (-0.063, 0.137) |
| TR7 | 0.021 | (-0.057, 0.099) | 0.016 | (-0.075, 0.107) |
| TR8 | 0.019 | (-0.057, 0.095) | 0.035 | (-0.061, 0.131) |
| TR9 | 0.016 | (-0.055, 0.086) | 0.031 | (-0.057, 0.120) |
| TR10 | 0.012 | (-0.052, 0.086) | 0.027 | (-0.068, 0.121) |
| TR11 | 0.018 | (-0.061, 0.096) | 0.034 | (-0.066, 0.133) |
| TR12 | 0.023 | (-0.055, 0.101) | 0.039 | (-0.058, 0.136) |

**Supplemental Figure S1: Technical Replicate Differences.** Density plots of the difference in methylation (Beta value) between pairs of technical replicates for each platform (EPIC or 450K) for the two methods (SeSAMe in blue and SWAN in red). Each plot displays one of the twelve pairs of technical replicates..


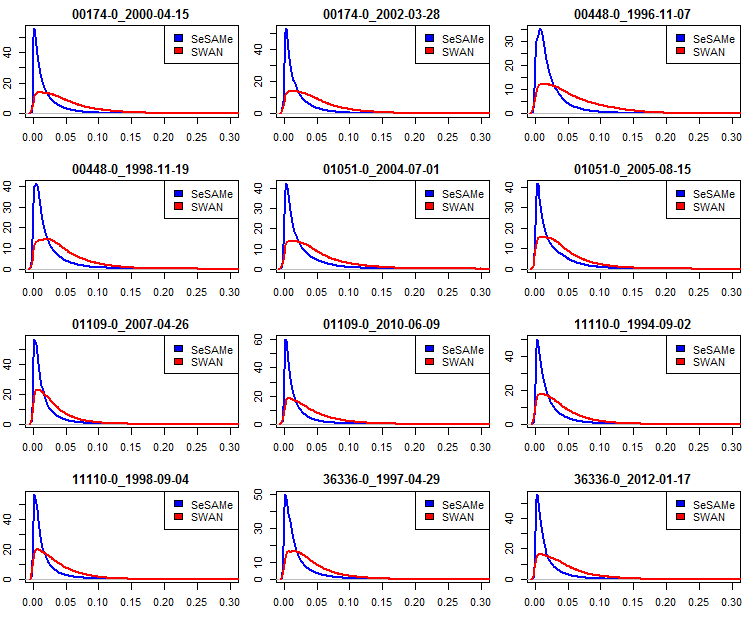


**Supplemental Figure S2. Batch Effect Adjustment.** A-B. Heatmaps of the statistical significance (-log10 p-values) of association between principal components and batch for the unadjusted raw normalized data, the RUVm adjusted data and ComBat adjusted data both the A. 450K and B. EPIC platforms. C-D. Heatmaps of the proportion PC variance (R-squared from the association model) explained by batch for the unadjusted raw normalized data, the RUVm adjusted data and the ComBat adjusted data in both the C. 450K and D. EPIC platforms.


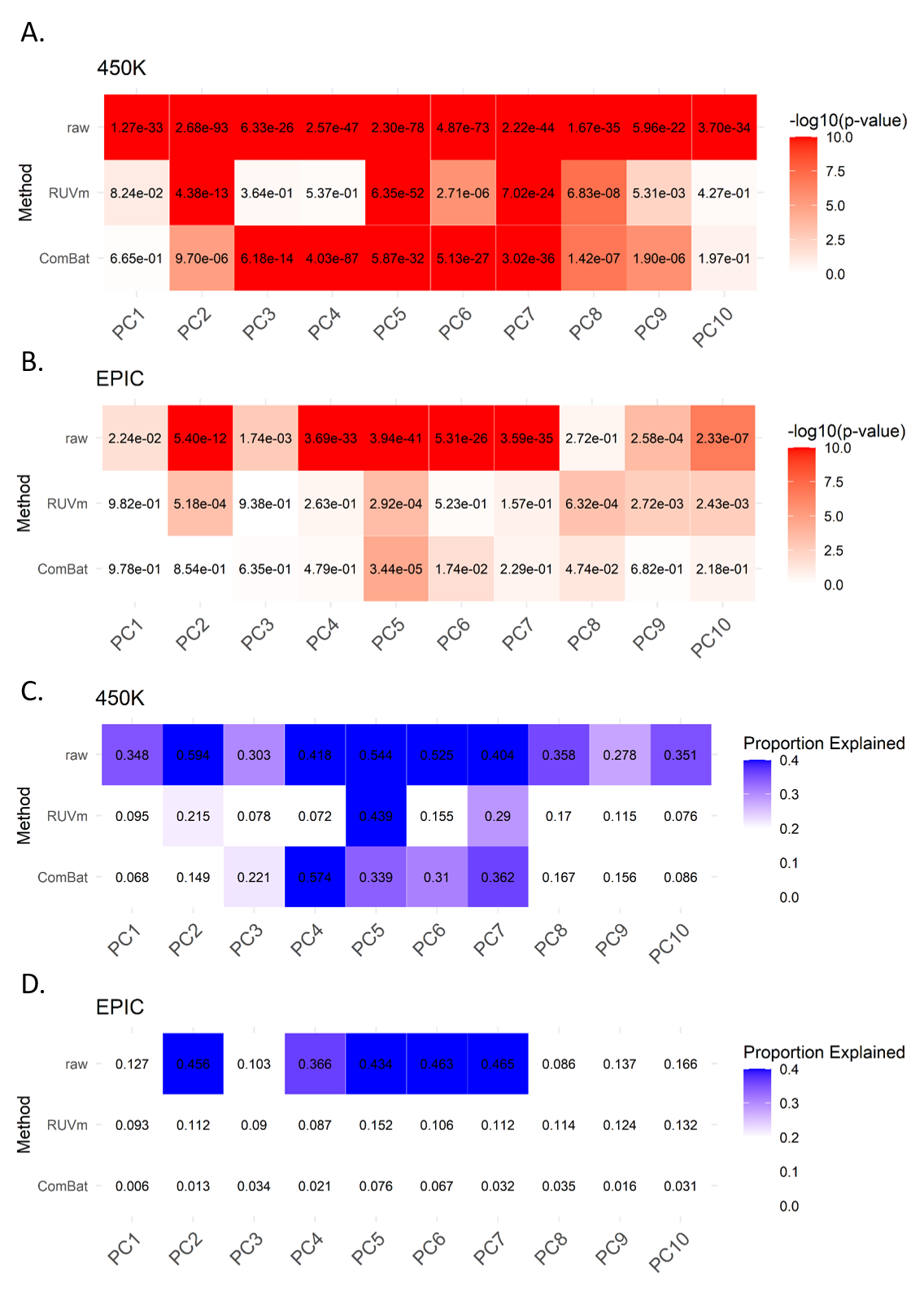


**Supplemental Figure S3. Mean Beta value for probes with no variability.** Histograms showing the mean Beta value for those probes which failed the range filter (Beta range < 0.05) are shown for the A. 450K and B. EPIC platforms. Histograms for the mean Beta value for those probes which passed the range filter (Beta range > 0.05) are shown for the C. 450 and D. EPIC platforms.


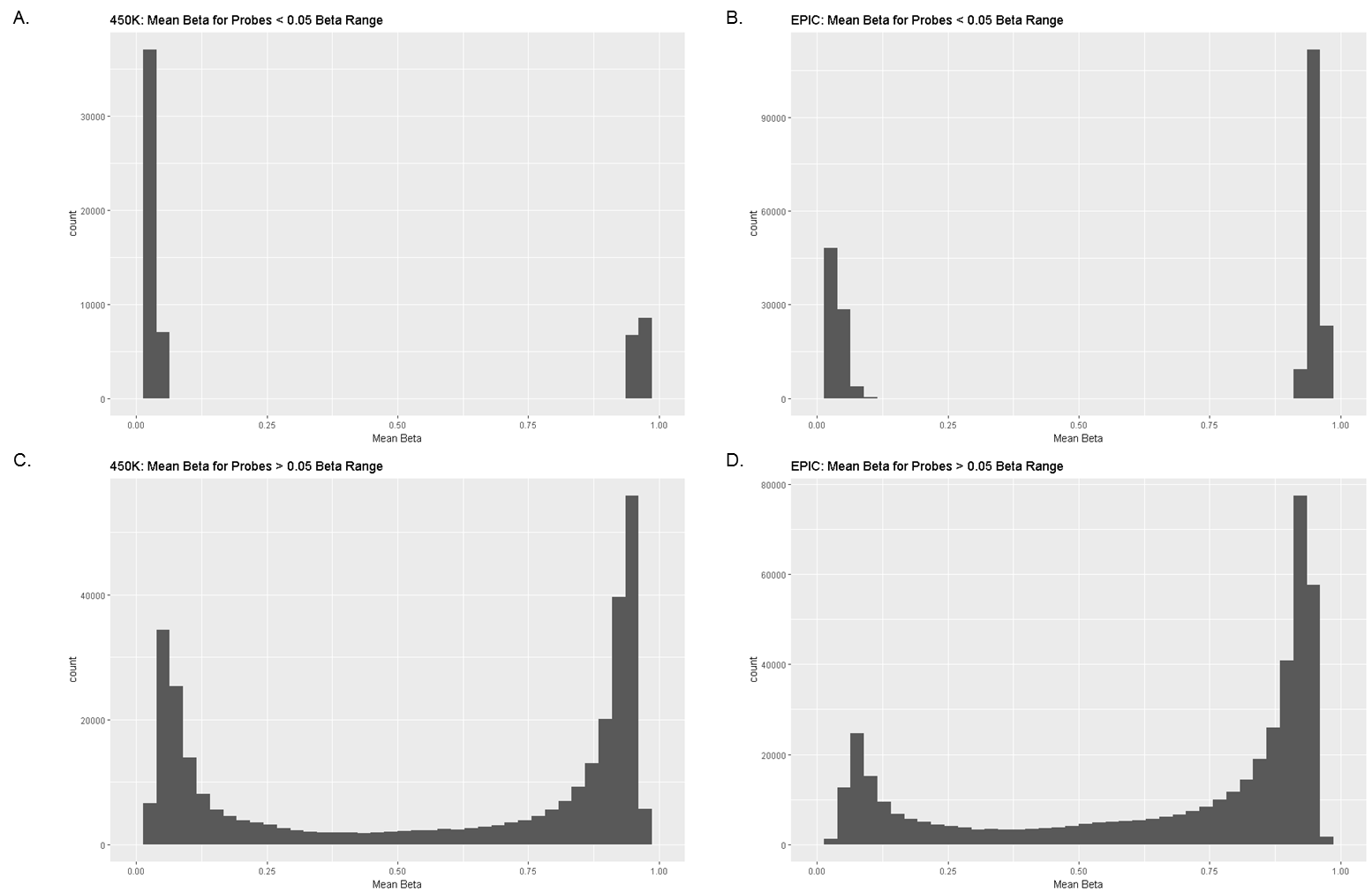


**Supplemental Figure S4. Effect of extra filtering by probe variability on genomic distribution of probes.** The proportion of probes on each chromosome is shown in the pre- and post- probe range filtering datasets in red and blue respectively (filtered probes with a Beta range < 0.05) for both the A. 450K and B. EPIC platforms.


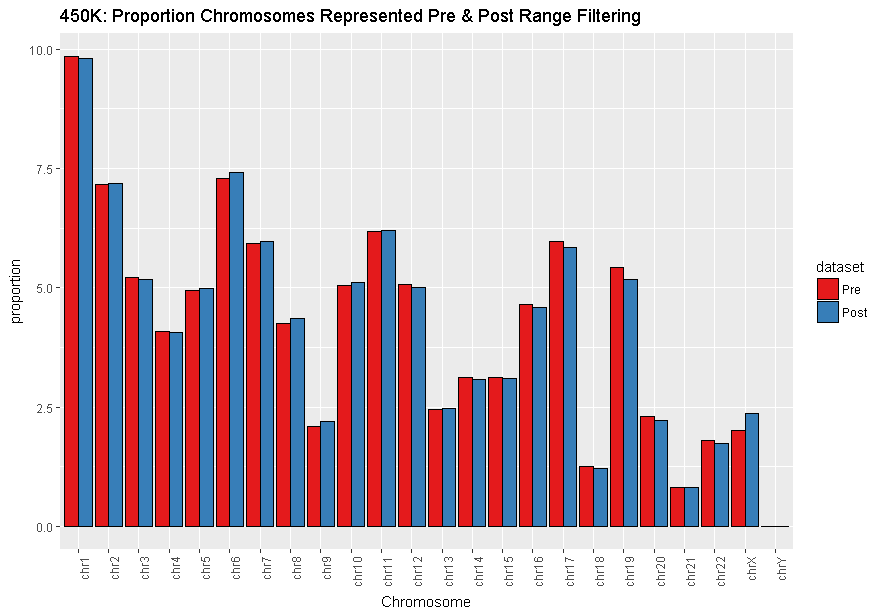

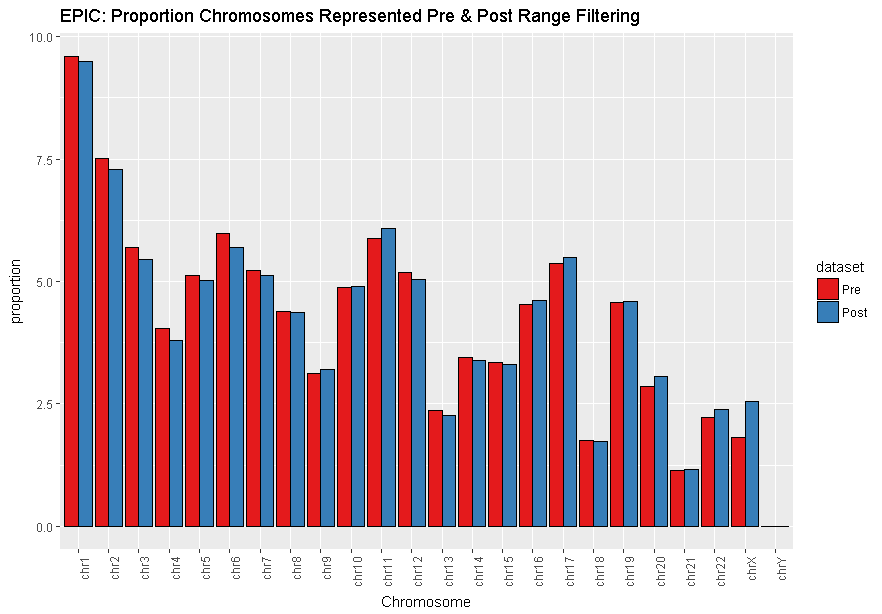


A.

B.

**Supplemental Figure S5. Technical Replicate Correlation and Beta Range.** Probe correlation coefficients from the technical replicates within the 450K (Eq-2) is plotted against the methylation Beta range.


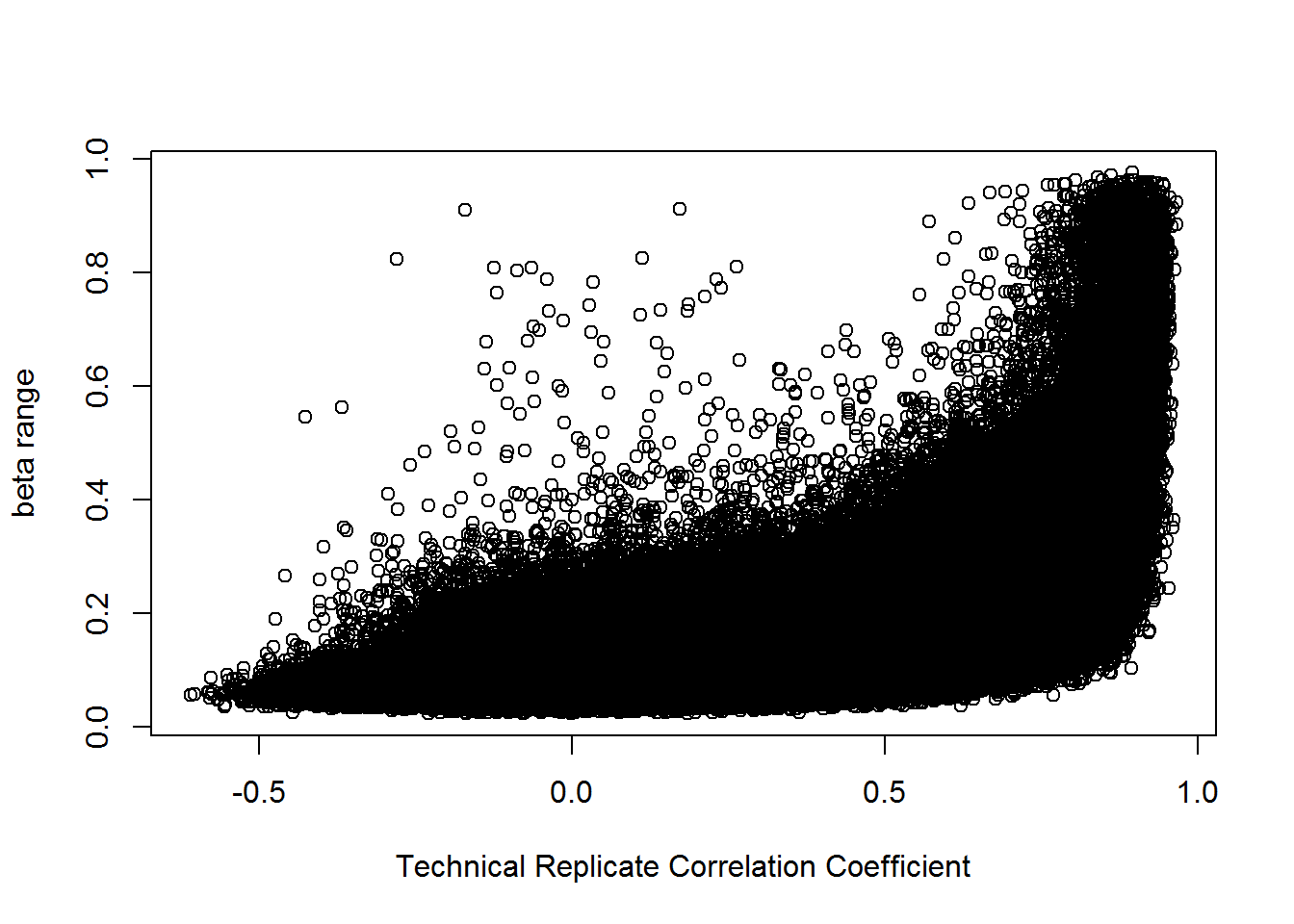


**Supplemental Figure S6. Genomic inflation factor across different datasets.** The qq-plots for the different datasets are shown for A. SWAN normalized 450K (lambda = 3.02), B. SWAN normalized EPIC (lambda = 0.83)., C. SeSAMe 450K (lambda = 0.93) and D. SeSAMe EPIC (lambda = 0.98). The blue dots are the observed p-values, while the black line shows the expected distribution these p-values should follow.


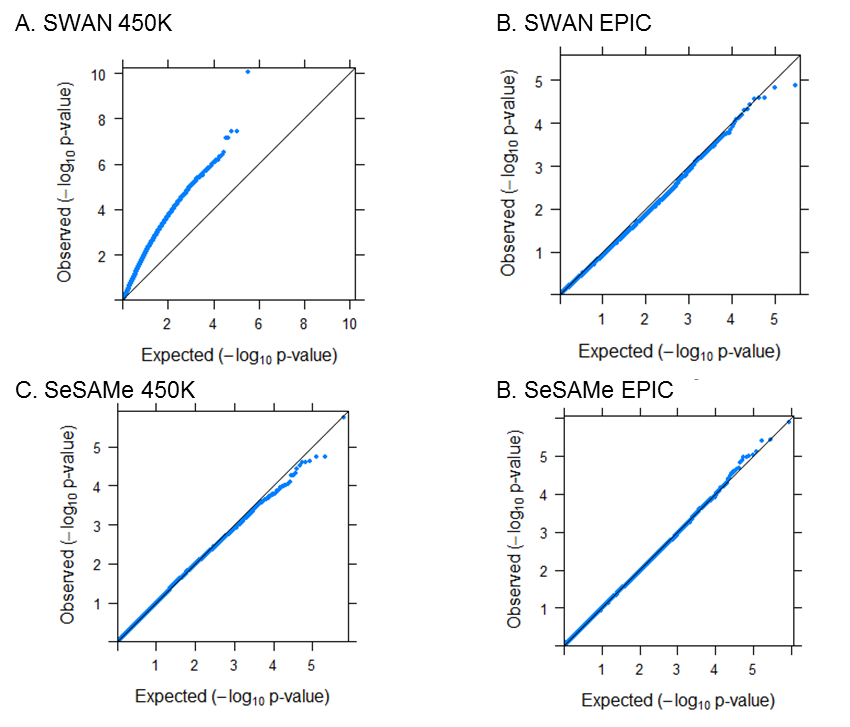


**Supplemental Figure S7. Final meta-analysis pipeline.** The final recommendations for a meta-analysis using the two Illumina methylation platforms. The blue box represents the raw data, the orange boxes represent each processing step, the gray boxes report how many probes are filtered out in each step and the green boxes are the final methylation candidates.


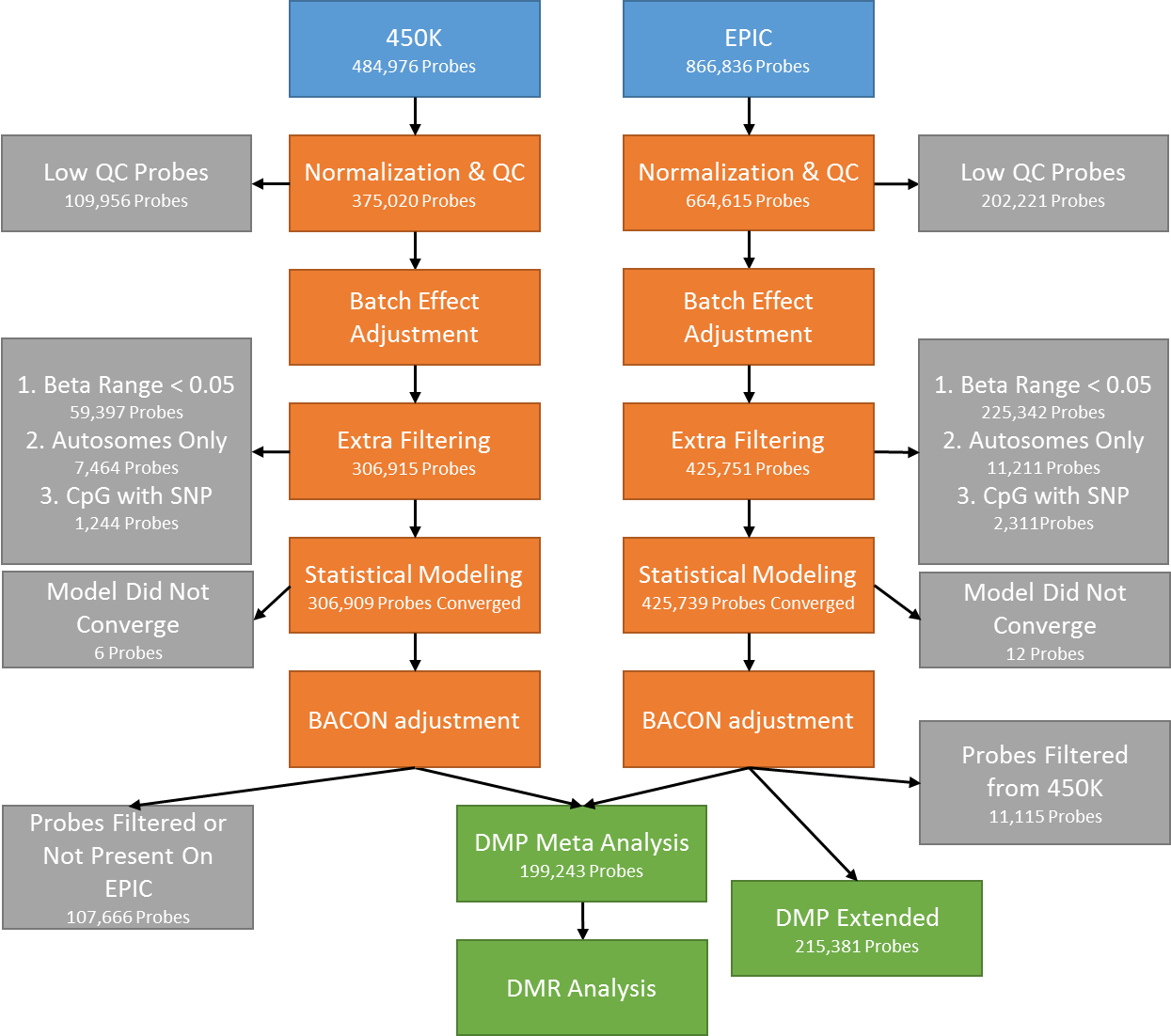

Supplement: Supplementary file 1 — Additional file 1: Table S1. PC Association with Platform. Principal component analysis was performed across the ssNoob normalized dataset (e.g. both the 450K and EPIC platforms normalized together) with and without batch effect adjustment using Combat. For the first 10 PCs, the percent variance explained and the p-value for representing the association between the PC and platform is reported. Associations with a p-value < 0.05 are highlighted in yellow. Figure S1. Technical Replicate Differences. Density plots of the difference in methylation (Beta value) between pairs of technical replicates for each platform (EPIC or 450K) for the two methods (SeSAMe in blue and SWAN in red). Each plot displays one of the twelve pairs of technical replicates. Figure S2. Batch Effect Adjustment. Heatmaps of the association between principal components and batch for both the unadjusted raw normalized data, the RUVm adjusted data and ComBat adjusted data in both the A. 450K and B. EPIC platforms. Figure S3. Mean Beta value for probes with no variability. Histograms showing the mean Beta value for those probes which failed the range filter (Beta range < 0.05) are shown for the A. 450K and B. EPIC platforms. Histograms for the mean Beta value for those probes which passed the range filter (Beta range > 0.05) are shown for the C. 450 and D. EPIC platforms. Figure S4. Effect of extra filtering by probe variability on genomic distribution of probes. The proportion of probes on each chromosome is shown in the pre- and post- probe range filtering datasets in red and blue respectively (filtered probes with a Beta range < 0.05) for both the A. 450 K and B. EPIC platforms. Figure S5. Technical Replicate Correlation and Beta Range. Probe correlation coefficients from the technical replicates within the 450 K (Eq-2) is plotted against the methylation Beta range. Figure S6. Genomic inflation factor across different datasets. The qq-plots for the different datasets are shown for A. SWAN normalize [file 13104_2021_5741_MOESM1_ESM.docx]
